# Red cell distribution width, neutrophil lymphocyte ratio and interleukin 10 are good prognostic markers in multiple myeloma

**DOI:** 10.37796/2211-8039.1405

**Published:** 2023-06-01

**Authors:** Marwa M. Seyam, Noha E. Esheba, Manal A. Eid, Mamdouh A. Gabr

**Affiliations:** a Internal Medicine Departments, Faculty of Medicine, Helwam University, Cairo, Egypt; b Internal Medicine Departments, Faculty of Medicine, Tanta University, Tanta, Egypt; c Clinical Pathology Departments, Faculty of Medicine, Tanta University, Tanta, Egypt

**Keywords:** ***Keywords:*** Interleukin 10, Neutrophil lymphocyte ratio, Red cell distribution width, Multiple myeloma

## Abstract

**Background:**

Multiple myeloma (MM) is still an incurable disease so we need to continue developing new diagnostic and prognostic options for its management. There are multiple prognostic factors for MM, but most of them are costly and time consuming. Hence comes the urge to identify bed side and low cost prognostic tools, that is why this study was aiming to identify in Egyptian MM patients.

**Materials and methods:**

The study was carried on 60 newly diagnosed multiple myeloma patients and 20 age and sex matched healthy individuals as controls. Studied subjects were subdivided into two groups: Group I: 60 multiple myeloma patients which were subdivided into three subgroups: Stage I: 10 patients, Stage II: 17 patients, Stage III: 33 patients, Group II: 20 healthy controls.

**Results:**

A progressive significant increase in IL-10, RDW, NLR, and beta2 microglobulin (β2M) with disease progression from stage I towards stage III as compared to the control group. However, IL-10, RDW, and NLR have the best prognostic efficiency value regarding to sensitivity, specificity and positive predictive value when compared with β2M.

**Conclusions:**

IL-10, RDW, and NLR are simple, easy and bedside tests (in the case of RDW, and NLR). They have high sensitivity and specificity when compared to β2M, which is a well-established prognostic factor that highlights the valuable role they play as prognostic markers in MM.

## 1. Introduction

MM is a malignant plasma cell neoplasm formed from a single clonal growth in the bone marrow [1], accounting for around 10% of all hematologic malignancies [2].

The most common symptoms are fatigue, unexplained weight loss, anaemia, bone discomfort or fractures, poor renal function, coagulation abnormalities, neurologic symptoms, and blood hyper-viscosity [3]. However, because myeloma patients’ clinical development and survival are so unpredictable, we can’t give an exact prognosis based on their condition at the time of diagnosis. Furthermore, bone marrow biopsy is invasive, and several additional clinical tests, such as fluorescence in situ hybridization (FISH), are too expensive. As a result, researchers are focusing their efforts on merging some patient-related factors in order to develop new prognostic models.

Systemic inflammation has been identified as a key factor in growth of tumour. In this context, C-reactive protein, albumin, as well as NLR have been investigated in several studies as effective markers to measure the correlation between inflammation and survival of various cancer patients [4]. NLR, which is neutrophil count (cells/L) divided by lymphocyte count (cells/L), has recently been discovered to be a prognostic factor for both solid tumours and hematologic malignancies [5].

The degree of variability in erythrocyte volume, also known as anisocytosis, is reflected by RDW, which is typically assessed in the complete blood cell count test. It is easily estimated by dividing the erythrocyte standard deviation by the mean corpuscular volume (MCV) of red blood cells (RBCs) [6].

MM cells and the bone marrow microenvironment induce autocrine or paracrine secretions of several mediators. Several mediators play essential roles in the MM angiogenic process, leading to tumour growth, invasion, and metastasis, according to extensive study; for example, increased levels of Interleukin-16 (IL-16) and IL-17 result in a poor MM prognosis [7].

IL-10 is an inflammatory cytokine that is primarily produced by myeloma-associated macrophages [8] and plays a key role in B cell proliferation [9] and plasma cell differentiation. IL-10 has been linked to the immune suppressive microenvironment in MM and disease progression [10]. However, it’s unclear how IL-10 levels in the blood connect with different phases of MM and clinical symptoms.

## 2. Materials and methods

The study was carried on 60 newly diagnosed MM patients based on clinical manifestations and investigations and 20 age and sex matched healthy individuals as controls recruited from the wards of Hematology unit, Internal Medicine Department, Tanta University Hospital from December 2018 to December 2020. Our work was approved by the Medical Ethical Committee of faculty of Medicine, Tanta University, approval number (32823/01/19).

Studied subjects were subdivided into two groups: Group I: 60 multiple myeloma patients and Group II: 20 healthy controls. Group I patients were further subdivided according to the International Staging System (ISS) [11] into three subgroups Stage I: 10 patients, Stage II: 17 patients, Stage III: 33 patients. Patients who newly diagnosed with MM were included in the study. On the other hand, patients with any of the following were excluded from the study: history of smoking, history of previous inflammatory disease such as infections and collagenvascular disease, previous treatment for MM, history of chronic disease like diabetes mellitus and liver disease, history of non-steroidal anti-inflammatory drugs (NSAIDs) intake and history of any malignant disease. All patients and controls were subjected to: full history taking, full clinical examination, laboratory investigations including: urine analysis, complete blood count with estimation of RDW and NLR, blood urea and serum creatinine, serum albumin, serum calcium, erythrocyte sedimentation rate (ESR), serum lactate dehydrogenase (LDH), serum protein electrophoresis (for patient only), bone marrow aspiration and biopsy (for patient only), immunofixation (for patient only), serum β2M, serum IL-10 estimation.

### 2.1. Blood sampling

10 ml venous blood samples were collected from each patient and control. 2 ml blood were introduced into EDTA tube for complete blood count (CBC). 2 ml blood for ESR. About 6 ml blood were introduced into plain tubes and left into water bath for 30 min and then serum samples were collected after centrifugation for estimation of creatinine, urea, calcium, β2M, albumin and LDH. Aliquots of serum samples were stored at −20 °C for further estimation of IL-10 with avoided repeated freeze–thaw cycles.

### 2.2. Interleukin 10 in serum by ELISA

The microplate has been pre-coated with an anti-IL-10 antibody. Pipette standards and samples into the wells, and the immobilized antibody will be absolutely reactive to any IL-10 present. After removing any unbound substances, IL-10 antibody, which could be biotin-conjugated, is added. When an antibody-antigen-enzyme-labeled antibody complex is made, avidin conjugated HRP is added after washing, the TMB substrate solution is added to the wells to remove any unbound HRP-avidin, and the colour changes to blue. When a stop solution is added to the wells, the colour changes from blue to yellow. The intensity of the colour is in proportion to the amount of IL-10 bound from the start.

### 2.3. Statistical analysis

We used SPSS version 22 in the data analysis. The data were presented as mean, and standard deviation (SD) and were analyzed by Chi-square test (x2), Independent t-test, Mann–Whitney U test, ANOVA (f) test, Kruskal Wallis test, Spearman correlation (rs), and Receiver Operating Characteristic (ROC). The diagnostic cutoff points were determined based on the cutoff value with the highest accuracy. At the level of 0.05, the P-value was considered significant.

## 3. Results

Comparison between patients in different disease stages and control showed no significant difference in gender and age. While serum creatinine rose as the disease stage progressed, that the difference was statistically significant in stages II and III when compared to stage I and controls. ESR, LDH and serum calcium as well rose as the disease stage progressed, that the difference was statistically significant in all the stages when compared to controls. Same results were noticed for IL-10, RDW%, NLR and β2M. The levels of M protein were significantly increasing as the disease stage progressed, while this was not noticed for plasma cell percentage in the bone marrow (Table 1, Table 4).

On performing correlation test, we found significant positive correlations between IL-10 and all of the studied parameters except for hemoglobin and serum albumin which showed significant negative correlations. The same results were found for RDW % and NLR except that both did not show correlation with M protein level (Table 2).

**Fig. 1:**
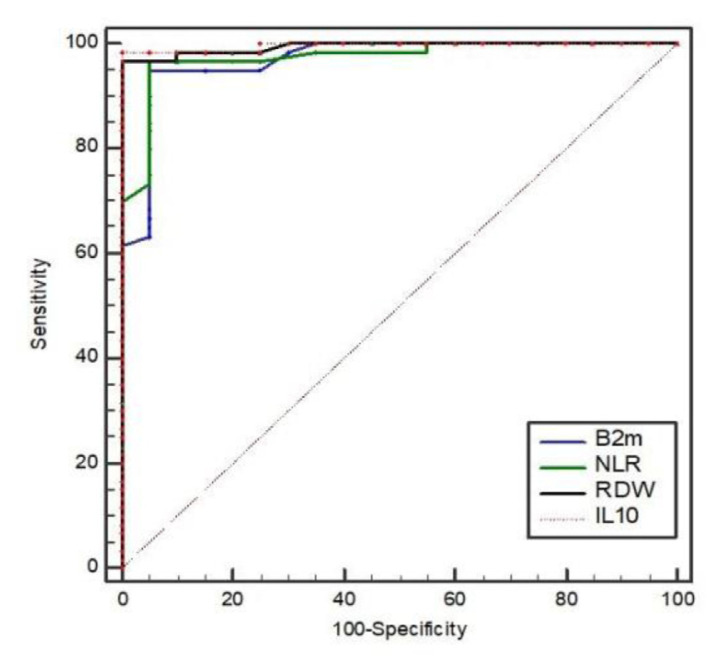
ROC curve for IL-10, RDW, NLR and β2M.

On analyzing the results of the ROC curve we found that both IL-10 and RDW% had superior sensitivity and specificity in relation to β2M, while NLR had higher sensitivity and similar specificity to β2M (Table 3, Fig. 1).

## 4. Discussion

There is a rising need to establish simple, easy and cheap markers of prognosis in MM. That is why we performed this work to evaluate the prognostic role of RDW%, NLR and IL-10 in Egyptian multiple myeloma patients. This work was performed on 60 Egyptian MM patients who were divided according to the disease stage into 3 subgroups: stage I, II and III. Twenty healthy controls were also included.

Regarding to IL-10, it was significantly higher in MM patients compared to the control group. Additionally, a progressive significant increase in the level of IL-10 was observed among MM patients as we proceed from stage I towards stage III, a finding that indicates a possible prognostic role of IL-10 in MM. This was in agreement with Shekarriz R et al., 2018 [12] who conducted a study on 40 patients with symptomatic MM and found that serum IL-10 levels were increasing with advanced stages of disease and mean serum IL-10 levels in MM patients with stage III were significantly higher than those of stage I and of stage II patients.

Regarding to the results of RDW, it was significantly higher in stage III when compared to stages I and II and control. Also RDW was significantly higher in stage I and II when compared to control, Which suggests that high RDW carries a bad prognosis in MM. This was in agreement with Zhou D et al. (2018) [13] who conducted a study on 162 patients with MM and found that RDW was significantly increased when the disease progressed.

Regarding to the results of NLR, it was significantly higher in stage III when compared to stages I and II and control. Also NLR was significantly higher in stage I and II when compared to control. This was in agreement with Mu S et al., 2018 [14] who conducted a study on MM patients and the results indicated that elevated pretreatment NLR was significantly associated with advanced tumor stages.

Regarding to the results of β2M, there was highly significant increase in β2M in the three disease stages when compared to the control. This was in agreement with Shekarriz R et al., 2018 [12] who conducted a study on 40 patients with symptomatic MM and found that β2M value in stage III patients was significantly higher than those with stage II and stage I.

In our results, IL-10 showed significant positive correlation with RDW, NLR, β2M, Plasma cells, M protein, creatinine, total calcium, ESR and LDH (P < 0.001) and significant negative correlation with Hb and albumin (P < 0.001). In partial agreement with the previous results, Shekarriz R et al., 2018 [12] found that, there is a positive and significant correlation between IL-10 levels and β2M. Positive correlation was observed between IL-10 levels and plasma cells. However, they didn’t observe any significant correlation between IL-10 concentration with other parameters such as M protein, total calcium, Hb and ESR levels.

In our results, RDW showed significant positive correlation with IL-10, NLR, β2M, Plasma cells, creatinine, total calcium, ESR and LDH (P < 0.001) except non significant correlation with M protein and significant negative correlation with Hb and albumin (P < 0.001) this was in agreement with Meng S et al., 2017 [15] who found that there were significant correlations between RDW and these parameters (ESR, LDH, creatinine, total calcium, albumin and Plasma cells) except M protein that did not show any significant correlation with it.

In our results, NLR showed significant positive correlation with IL-10, RDW, β2M, Plasma cells, creatinine, ESR and LDH (P < 0.001) except non significant correlation with M protein and significant negative correlation with Hb, total calcium and albumin (P < 0.001). This was partially in agreement with Shi L et al., 2017 [16] who found that there were significant correlations between NLR, β2M, and LDH.

Regarding to the results of serum IL-10 in our study, the best cut off level was >1900 pg/ml, at this level, the diagnostic sensitivity was 98.33%, the diagnostic specificity was 100% and the positive predictive value was 100%. The best cut off level of serum RDW was >13**%**, at this level the diagnostic sensitivity was 96.67%, the diagnostic specificity was 100% and the positive predictive value was 100%. The best cut off level of NLR was >0.9**%**, at this level the diagnostic sensitivity was 96.67%, the diagnostic specificity was 95% and the positive predictive value was 98.3%. The best cut off level of serum β2M was >2.1**%**, at this level the diagnostic sensitivity was 95%, the diagnostic specificity was 95% and the positive predictive value was 86.4%. IL-10 revealed the highest sensitivity and specificity among the four studied parameters. To our knowledge, no other work addressed our three studied parameters (IL-10, RDW%, NLR) in comparison to the β2M (a known prognostic marker in MM).

## Figures and Tables

**Table 1:** Comparison between patients in different disease stages and control regarding to demographic data and some studied parameters.

Parameter		MM patients			Controls	P value

		Stage I	Stage II	Stage III		
Male/Female	N (%)	6/4 (60/40)	9/8 (53/47)	16/17 (48/52)	10/10 (50/50)	0.93
Age (years)	Mean ± SD	53.90 ± 7.31	53.94 ± 9.58	57.79 ± 9.59	53.30 ± 8.92	0.27
Hemoglobin (gm/dl)	Mean ± SD	9.45 ± 0.92	8.47 ± 1.17	8.20 ± 1.37	13.35 ± 1.30	<0.001
		P1^g^ < 0.001 P2^h^ < 0.001 P3^i^ < 0.001 P4^j^ = 0.06 P5^k^ = 0.011 P6^l^ = 0.26				
Creatinine (mg/dl)	Mean ± SD	0.73 ± 0.24	1.55 ± 1.27	2.37 ± 1.74	0.69 ± 0.22	<0.001
		P1^g^ = 0.739 P2^h^ < 0.001 P3^i^ < 0.001 P4^j^ = 0.005 P5^k^ < 0.001 P6^l^ = 0.054				
ESR 1st hour^a^ (mm)	Mean ± SD	69.70 ± 16.66	77.76 ± 19.01	91.79 ± 31.6	18.40 ± 3.88	<0.001
		P1^g^ < 0.001 P2^h^ < 0.001 P3^i^ < 0.001 P4^j^ = 0.857 P5^k^ = 0.080 P6^l^ = 0.256				
LDH^b^ (u/l)	Mean ± SD	348.2 ± 58.23	352.76 ± 122.15	425.7 ± 336.6	158.2 ± 29.69	<0.001
		P1^g^ < 0.001 P2^h^ < 0.001 P3^i^ < 0.001 P4^j^ = 0.086 P5^k^ = 0.069 P6^l^ = 0.08				
Total calcium (mg/dl)	Mean ± SD	10.15 ± 0.79	11.05 ± 1.83	11.26 ± 1.94	8.89 ± 0.52	<0.001
		P1^g^ < 0.001 P2^h^ < 0.001 P3^i^ < 0.001 P4^j^ = 0.257 P5^k^ = 0.09 P6^l^ = 0.0.89				
IL-10^c^ (pg/ml)	Mean ± SD	2396.3 ± 143.39	3379.82 ± 754.3	4692 ± 545.9	671.±352.7	<0.001
		P1^g^ < 0.001 P2^h^ < 0.001 P3^i^ < 0.001 P4^j^ < 0.001 P5^k^ < 0.001 P6^l^ < 0.001				
RDW^d^%	Mean ± SD	14.33 ± 1.09	14.89 ± 1.21	17.77 ± 1.37	11.89 ± 0.59	<0.001
		P1^g^ < 0.001 P2^h^ < 0.001 P3^i^ < 0.001 P4^j^ = 0.497 P5^k^ < 0.001 P6^l^ < 0.001				
NLR^e^%	Mean ± SD	2.86 ± 4.29	2.90 ± 0.49	4.89 ± 1.51	0.77 ± 0.52	<0.001
		P1^g^ < 0.001 P2^h^ < 0.001 P3^i^ < 0.001 P4^j^ = 0.003 P5^k^ < 0.001 P6^l^ < 0.001				
β2M^f^ (mg/l)	Mean ± SD	2.63 ± 0.60	4.54 ± 0.54	39.60 ± 36.24	1.61 ± 0.92	<0.001
		P1^g^ = 0.001 P2^h^ < 0.001 P3^i^ < 0.001 P4^j^ < 0.001 P5^k^ < 0.001 P6^l^ < 0.001				
M protein (mg/dl)	Mean ± SD	2495.6 ± 2586.9	2180.1 ± 1517.1	3425.2 ± 2349.68	NA f	0.001
		P4^j^ < 0.001 P5^k^ < 0.001 P6^l^ < 0.001				
Plasma cell %	Mean ± SD	26.50 ± 12.40	31.53 ± 21.76	54 ± 24.95	NA^m^	0.11

a erythrocyte sedimentation rate.

b lactic dehydrogenase.

c interleukin 10.

d red cell distribution width percentage.

e neutrophil lymphocyte ratio.

F beta 2 microglobulin.

g comparison between stage I patients and controls.

h comparison between stage II patients and controls.

i comparison between stage III patients and controls.

j comparison between stage I patients and stage II patients.

k comparison between stage I patients and stage III patients.

l comparison between stage II patients and stage III patients.

m not applicable.

**Table 2:** Correlation of IL-10, RDW% and NLR with the different studied parameters.

Parameters	IL-10 (pg/ml)		RDW %		NLR %	
		
	r_s_^a^	P-Value	r_s_^a^	P-Value	r_s_^a^	P-Value
RDW %	0.82	<0.001	–	–	0.78	<0.001
NLR %	0.86	<0.001	0.78	<0.001	–	–
IL-10 (pg/ml)	–	–	0.82	<0.001	0.86	<0.001
β2M (mg/l)	0.86	<0.001	0.841	<0.001	0.84	<0.001
Plasma cell %	0.34	0.007	0.54	<0.001	0.47	<0.001
M protein (mg/dl)	0.295	0.02	0.21	0.10	0.19	0.13
Creatinine (mg/dl)	0.55	<0.001	0.51	<0.001	0.57	<0.001
Total calcium (mg/dl)	0.49	<0.001	0.42	<0.001	0.48	<0.001
Hb (g/dl)	−0.63	<0.001	−0.61	<0.001	−0.63	<0.001
ESR (mm)	0.63	<0.001	0.66	<0.001	0.65	<0.001
LDH (u/l)	0.40	<0.001	0.57	<0.001	0.48	<0.001
Albumin (g/dl)	−0.56	<0.001	−0.52	<0.001	−0.55	<0.001

a Spearman correlation.

**Table 3:** Interpreting diagnostic tests.

Validity	IL-10 (pg/ml)	RDW %	NLR %	β2M (mg/l)
Area under curve	0.996	0.994	0.973	**0.969**
Cut off value	>1900	>13	>0.9	**>2.1**
Sensitivity	98.33%	96.67%	96.67%	**95%**
Specificity	100%	100%	95%	**95%**
PPV^a^	100%	100%	98.3%	**98.3%**
NPV^b^	95.2%	90.9%	90.5	**86.4%**

a positive predictive value.

b negative predictive value.

**Table 4:** Univariate and multivariate logistic regression model of some studied parameters as predictors for multiple myeloma.

	Univariate		Multivariate	
	
	OR^a^ (95%C.I)^b^	P value	OR^a^ (95%C.I)^b^	P value
Age (years)	1.034 (0.976–1.094)	0.24	–	–
Sex	0.935 (0.340–2.574)	0.89	–	–
IL-10 (pg/ml)	1.004 (1.002–1.006)	0.001	1.0037 (1.0016–1.0058)	<0.001
RDW%	30.232 (2.939–310.999)	0.004	30.232 (2.939–310.999)	0.004
NLR%	20.150 (3.842–105.676)	<0.001	20.150 (3.842–105.676)	<0.001
β2m (mg/l)	6.759 (2.348–19.454)	<0.001	6.064 (1.8552–19.8238)	0.002
Hb (g/dl)	−0.040 (0.003–0.501)	0.01	−0.040 (0.003–0.501)	0.01
Creatinine (mg/dl)	21.605 (2.905–160.665)	0.003	21.605 (2.905–160.665)	0.003
Total calcium (mg/dl)	7.054 (2.235–22.263)	<0.001	48.162 (1.852–1251.974)	0.01
ESR (mm)	1.827 (0.749–4.451)	0.18	–	–
LDH (u/l)	1.031 (1.014–1.049)	<0.001	1.051 (1.012–1.093)	0.009
Albumin (g/dl)	(0.0248–0.2798) 0.083 -	<0.001	−0.020 (0.001–0.343)	0.006

a Odds ratio.

b 95% confidence interval.
